# The effect of strut thickness on shear stress distribution in a preclinical model

**DOI:** 10.1007/s10554-017-1173-4

**Published:** 2017-05-31

**Authors:** Erhan Tenekecioglu, Ryo Torii, Christos Bourantas, Yosuke Miyazaki, Carlos Collet, Rasha Al-Lameé, Kadem Al-Lameé, Yoshinobu Onuma, Patrick W. Serruys

**Affiliations:** 1 Department of Cardiology, Erasmus University Medical Centre, Thoraxcenter, Rotterdam, The Netherlands; 2 Department of Mechanical Engineering, University College London, London, UK; 3 Department of Cardiology, University College of London Hospitals, London, UK; 4 Barts Heart Centre, Barts Health NHS Trust, London, UK; 5 Department of Cardiology, Academic Medical Center, University of Amsterdam, Amsterdam, The Netherlands; 6 Imperial College London, London, UK; 7 Arterius, Leeds, UK; 8 University of Melbourne, Melbourne, Australia

**Keywords:** Bioresorbable scaffold, Local hemodynamics, Endothelial shear stress, Strut design

## Case report

In-vitro and in-silico studies have shown that stent design and struts thickness introduce significant changes in local hemodynamics. The protruding struts disrupt the laminar flow with the impacts of strut thickness and strut shape. Angiographic and optical coherence tomography (OCT) data were implemented to reconstruct three-dimensional (3D) geometry of the right coronary artery (RCA) of a healthy mini-swine implanted with 3.0 × 14 mm ArterioSorb (Arterius, UK) with 95 micron (µm) strut thickness in proximal segment (final post-dilatation mean lumen diameter: 2.78 mm) and 3.0 × 14 mm ArterioSorb with 120 µm strut thickness in mid-segment (final post-dilatation mean lumen diameter: 2.80 mm) of the vessel.

**Fig. 1:**
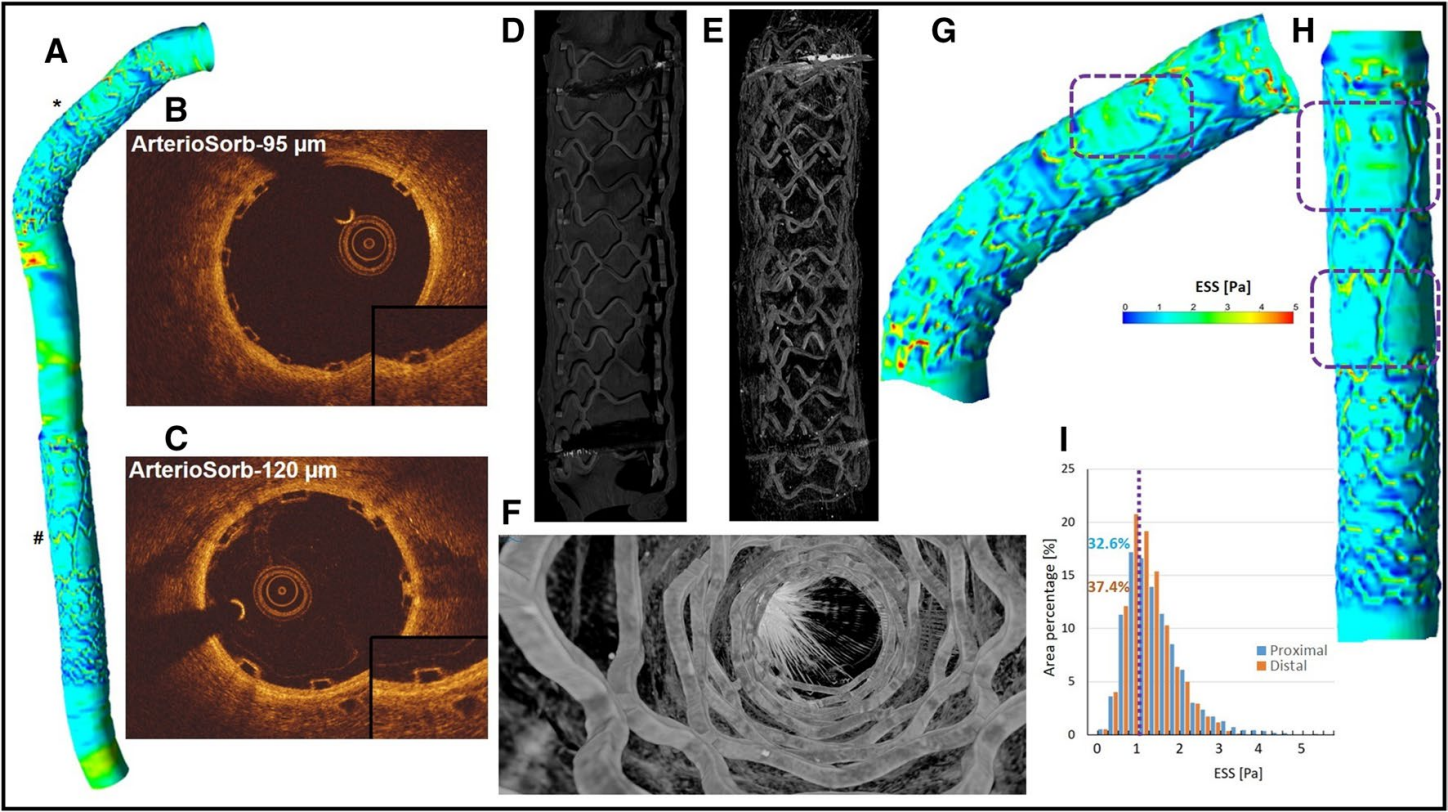
The CFD model of the two ArterioSorb scaffolds (*ArterioSorb-95 µm, ^**#**^ArterioSorb-120 µm) with different strut thicknesses in RCA (**a**). OCT demonstrates well apposed struts in ArterioSorb-95 µm (**b**) and in ArterioSorb-120 µm (**c**). Micro-computed tomography shows the scaffold architectures without any discontinuity in ArterioSorb-95 µm (**d**) and in ArterioSorb-120 µm (**e, f**). In proximal (**g**) and the distal scaffolds (**h**), there are wide zones with smooth surfaces (*dotted* zones) which are considered to be the result of cardiac motion during the OCT pullback. The histograms (**i**) demonstrate the percentages of the vessel surface exposed to low-ESS (<1 Pa) in scaffolded vessel segments

During computational fluid dynamic (CFD) study, endothelial shear stress (ESS) was quantified in the scaffolded segment around the circumference of the lumen per 5°-subunit and along the axial direction per 200 µm interval [1]. Median ESS was lower in the distal scaffold (ArterioSorb-120 µm) [1.17 (0.78–1.55) Pa] than in the proximal scaffold (ArterioSorb-95 µm) [1.25 (0.92–1.88) Pa] in Newtonian steady flow simulation (p < 0.0001). 37.4% of the scaffolded surface in ArterioSorb-120 µm and 32.6% in ArterioSorb-95 µm was exposed to a low (<1 Pa) athero-promoting ESS (Fig. 1). The p-value is based on 5°-subunit (n = 15,984) analysis using mixed effects regression model.

The difference in ESS may stem from strut thickness, luminal diameter, vessel curvature and flow boundary conditions. In the present case, after excluding other factors, lower ESS in ArterioSorb-120 µm is presumably ascribed to the thicker struts (Fig. 1). However, even in ArterioSorb-120 µm, the shear stress was not as low as reported in Absorb BVS (Median ESS:0.57 Pa) [2] which should be attributed to the thinner struts of ArterioSorb.
